# Agarwood Essential Oil Ameliorates Restrain Stress-Induced Anxiety and Depression by Inhibiting HPA Axis Hyperactivity

**DOI:** 10.3390/ijms19113468

**Published:** 2018-11-05

**Authors:** Shuai Wang, Canhong Wang, Zhangxin Yu, Chongming Wu, Deqian Peng, Xinmin Liu, Yangyang Liu, Yun Yang, Peng Guo, Jianhe Wei

**Affiliations:** 1 Key Laboratory of Bioactive Substances and Resources Utilization of Chinese Herbal Medicine, Institute of Medicinal Plant Development, Chinese Academy of Medical Sciences & Peking Union Medical College, Beijing 100193, China; zhuizhirun@163.com; 2 Ministry of Education & National Engineering Laboratory for Breeding of Endangered Medicinal Materials, Institute of Medicinal Plant Development, Chinese Academy of Medical Sciences & Peking Union Medical College, Beijing 100193, China; 3 Pharmacology and Toxicology Center, Institute of Medicinal Plant Development, Chinese Academy of Medical Sciences & Peking Union Medical College, Beijing 100193, China; cmwu@implad.ac.cn (C.W.); liuxinmin@hotmail.com (X.L.); 4 Hainan Provincial Key Laboratory of Resources Conservation and Development of Southern Medicine, Hainan Branch of the Institute of Medicinal Plant Development, Chinese Academy of Medical Sciences & Peking Union Medical College, Haikou 570311, China; xinzhuangjianpo@163.com (C.W.); yu_xin712@163.com (Z.Y.); yyliu@implad.ac.cn (Y.L.); yangyun43@gmail.com (Y.Y.); 5 Key Laboratory of State Administration of Traditional Chinese Medicine for Agarwood Sustainable Utilization, Hainan Branch of the Institute of Medicinal Plant Development, Chinese Academy of Medical Sciences & Peking Union Medical College, Haikou 570311, China; 6 School of Pharmacy, Hainan Medical College, Haikou 571199, China; pengdeqian2004@163.com

**Keywords:** agarwood essential oil, anti-anxiety, anti-depression, ethology, corticotropin releasing factor, HPA axis

## Abstract

In our previous investigation, we found that agarwood essential oil (AEO) has a sedative-hypnotic effect. Sedative-hypnotic drugs usually have an anxiolytic effect, where concomitant anxiety and depression are a common comorbidity. Therefore, this study further investigated the anxiolytic and antidepressant effects of AEO using a series of animal behavior tests on a restraint stress-induced mice model. The elevated plus maze (EPM) test, the light dark exploration (LDE) test, and the open field (OF) test demonstrated that AEO has a significant anxiolytic effect. Simultaneously, the tail suspension (TS) test and the forced swimming (FS) test illuminated that AEO has an antidepressant effect with the immobility time decreased. Stress can cause cytokine and nitric oxide (NO) elevation, and further lead to hypothalamic-pituitary-adrenal (HPA) axis hyperactivity. AEO was shown to dose-dependently inhibit the levels of cytokines, including interleukin 1α (IL-1α), IL-1β, and IL-6 in serum, significantly decrease the mRNA level of neural nitric oxide synthase (nNOS) in the cerebral cortex and hippocampus, and inhibit the nNOS protein level in the hippocampus. Concomitant measurements of the HPA axis upstream regulator corticotropin releasing factor (CRF) and its receptor CRFR found that AEO significantly decreases the gene expression of CRF, and significantly inhibits the gene transcription and protein expression of CRFR in the cerebral cortex and hippocampus. Additionally, AEO dose-dependently reduces the concentrations of adrenocorticotropic hormone (ACTH) and corticosterone (CORT) downstream of the HPA axis, as measured by ELISA kits. These results together demonstrate that AEO exerts anxiolytic and antidepressant effects which are related to the inhibition of CRF and hyperactivity of the HPA axis.

## 1. Introduction

Agarwood, a precious, fragrant, non-timber forest product of *Aquilaria* spp. (Thymelaeaceae), has been widely used for centuries in aromatic, incense, religious, aromatherapy, and medicinal preparations in Southeast Asia and the Middle East [1,2,3]. Medicinal application is one of its most important usages. In traditional medicine systems, agarwood is usually used for various medicinal purposes by a large number of peoples. It can relieve pain, arrest vomiting, and relieve asthma in traditional Chinese medicine [4]. In traditional Arabian medicine, agarwood has been widely used to treat digestive, neurodegenerative, and sedative diseases [5]. Additionally, modern pharmacological studies have shown that agarwood has many beneficial effects, including anti-inflammation [6,7,8], neuroprotection [9,10,11], and anti-depression effects [12,13]. Generally, agarwood essential oil (AEO) is considered to be the main active constituent of agarwood, possessing multiple pharmacological functions, especially on neural regulation. Our recent studies showed that AEO has a sedative-hypnotic effect with the potential mechanism of regulating the GABAergic system [14,15]. Hiroaki et al. also reported that vapor inhalation of AEO, where the main active constituents were benzylacetone, a-gurjunene, and (+)-calarene, was able to sedate mice [16]. Additionally, a number of compounds isolated from AEO have been demonstrated to have neural activity. For example, agarofuran was reported to have anxiolytic and antidepressant activity in mice [17]. Thus, a series of agarofuran-like derivatives were synthesized, and their activity was screened, among which buagafuran was identified as an effective compound for anti-anxiety with low toxicity and a high safety coefficient [17,18]. Buagafuran is a promising drug for the treatment of anxiety. Currently, phase II clinical trials of buagafuran on general anxiety are being conducted. Therefore, AEO might be an active constituent and a potential source of lead compounds for anxiety and depression.

Anxiety and depression disorders are some of the most prevalent psychiatric diseases in Western countries and they impose high personal and society costs [19]. A three-year multi-method study revealed that anxiety disorders had the highest 12-month prevalence estimates compared to all other psychiatric conditions in 30 European countries and a population of 514 million people [20]. Up to now, the pathogenesis mechanisms of anxiety and depression are not totally clear, and many factors contribute to the occurrence of anxiety and depression, including stress. Stress is a crucial inducing factor and usually leads to dysfunction of the neuroimmune-endocrine system. Hyperactivity of the hypothalamic-pituitary-adrenal (HPA) axis is accepted as one of the fundamental biological mechanisms that underlie psychiatric disorders, including anxiety and depression [21]. The HPA axis is often affected by many factors, such as cytokines and nitric oxide (NO). Cytokines, which are generally secreted by immune system cells, can also be synthesized and secreted by non-immune cells for the purpose of signaling neuroimmune cells. Proinflammatory cytokines, including interleukin-1α (IL-1α), IL-1β, and IL-6, are secreted in all components of the HPA axis and affect the secretion of corticotropin releasing factor (CRF) from the hypothalamus, adrenocorticotropic hormone (ACTH) from the pituitary, and glucocorticoids from the adrenal cortex [21]. NO, serving as a neurotransmitter in the brain, is generated by neuronal NO synthase (nNOS) and inducible NO synthase (iNOS) in brain and is involved in HPA axis regulation, particularly in response to CRF elevation [21]. CRF, broadly distributed in the central nervous system (CNS), is the major physiological regulator of the stress response and is one of the most studied neuropeptides in anxiety [22]. Most preclinical studies have focused on the CRF receptor (CRFR) as a result of the high expression density in regions of the brain that mediate anxiety and depression [23].

Aiming to validate the anxiolytic and antidepressant effects of AEO and explore the potential mechanism, we applied animal behavioral tests to assess the effects of AEO on restraint stress-induced anxiety in mice, and combined molecular biological technologies to explore the regulative effect of AEO on the HPA axis.

## 2. Results

### 2.1. Effects of AEO on Restraint Stress-Induced Anxiety by the Elevated Plus Maze (EPM) Test in Mice

**Figure 1:**
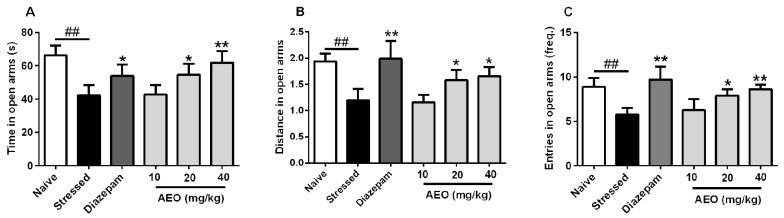
Effects of agarwood essential oil (AEO) on restraint stress-induced anxiety by the elevated plus maze (EPM) test. All mice except for the naive group received 10 days restraint stress, then (**A**) time, (**B**) distance, and (**C**) entries in open arms were recorded during a 5 min test period. Each value represents the mean ± SEM with *n* = 12, ^##^ *p* < 0.01 vs. the naive group, * *p* < 0.05, and ** *p* < 0.01 vs. the stressed group.

The elevated plus maze (EPM) test, one of the most popular behavioral tests, is frequently used on mouse anxiety model [24]. We applied the EPM test to assess the anxiolytic effect of AEO on restraint stress-induced anxiety in mice. As the results show in Figure 1, compared to the naive group, 10 days of repeated restraint stress (stressed group) significantly elicited anxiety in mice with the time, distance, and entries decreased in open arms. The positive control diazepam, a commonly used clinical anxiolytic drug, showed an anxiolytic effect on the time, distance, and entries in open arms, which all significantly increased. Treatment with AEO significantly and dose-dependently increased the time, distance, and entries in open arms, which efficiency (20 and 40 mg/kg) displayed comparable effects to diazepam (2.5 mg/kg).

### 2.2. Effects of AEO on Restraint Stress-Induced Anxiety by the Light Dark Exploration (LDE) Test in Mice

**Figure 2:**
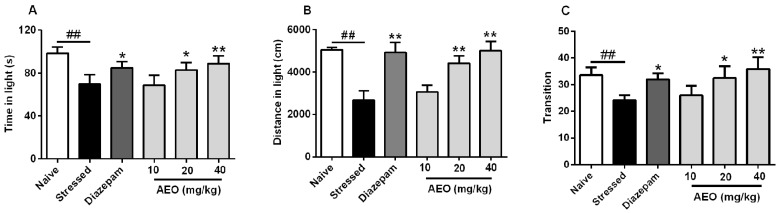
Effects of AEO on restraint stress-induced anxiety as shown by the light dark exploration (LDE) test. All mice except for the naive group received 10 days restraint stress, then (**A**) time, (**B**) distance, and (**C**) transition in light compartment were recorded during a 5 min test period. Each value represents the mean ± SEM with *n* = 12, ^##^ *p* < 0.01 vs. the naive group, * *p* < 0.05 and ** *p* < 0.01 vs. the stressed group.

The light dark exploration (LDE) test is another commonly used murine model of anxiety, which is based on the innate aversion of rodents to brightly illuminated areas, and on the spontaneous exploratory behavior of rodents in response to mild stressors, a novel environment, and light [25]. As the results show in Figure 2, repeated restraint stress (stressed group) led to significant decreases in time, distance, and transition in light compartment compared to the naive group. Diazepam (2.5 mg/kg) ameliorated the restraint stress-induced anxiety. Administration of AEO significantly increased the time and distance in light compartment of mice in a dose-dependent manner, as well as the elevation of transition to the light compartment. 40 mg/kg of AEO showed the best efficiency among the tested dosages, which effect is comparable to that of diazepam (2.5 mg/kg).

### 2.3. Effects of AEO on Restraint Stress-Induced Anxiety by the Open Field (OF) Test in Mice

**Figure 3:**
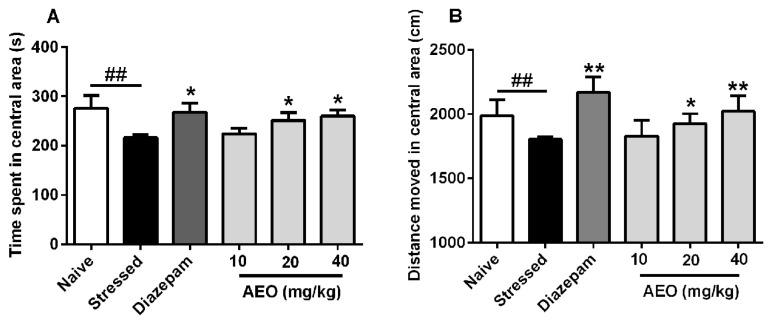
Effects of AEO on restraint stress-induced anxiety as assessed by the open field (OF) test. All mice except for the naive group received 10 days restraint stress, then (**A**) time spent and (**B**) distance moved in the central area were recorded over a 10 min session. Each value represents mean ± SEM with *n* = 12, ^##^ *p* < 0.01 vs. the naive group, * *p* < 0.05 and ** *p* < 0.01 vs. the stressed group.

In addition to the EPM and LDE tests, the open (OF) test is one of the most commonly used murine models of anxiety. We applied the OF test to assess the anxiolytic effect of AEO. As the results show in Figure 3, compared to the naive group, the stressed group showed significant decreases in the time and distance spent in the central area. Treatment with AEO (20 and 40 mg/kg) and diazepam (2.5 mg/kg) significantly increased the time and distance spent in the central area. The efficiency of AEO at the dose of 40 mg/kg was comparable to that of diazepam (2.5 mg/kg), whereas a dose of 10 mg/kg AEO did not have an obvious effect.

### 2.4. Effects of AEO on Restraint Stress-Induced Depression by the Tail Suspension (TS) Test in Mice

**Figure 4:**
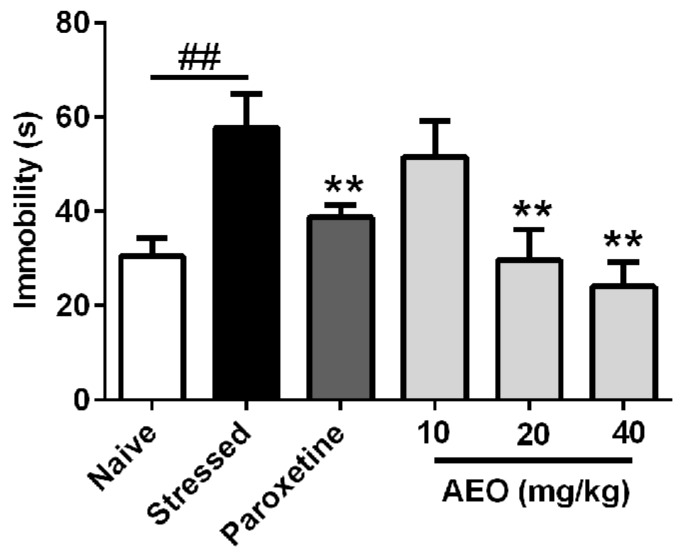
Effects of agarwood essential oil (AEO) on restraint stress-induced depression as assessed by the tail suspension (TS) test. All mice except for the naive group received 10 days restraint stress, and immobility was recorded during a 4 min test period. Each value represents mean ± SEM with *n* = 12, ^##^ *p* < 0.01 vs. the naive group, ** *p* < 0.01 vs. the stressed group.

In order to assess the anti-depressant effect of AEO, the tail suspension (TS) test was carried out to detect the immobility. As shown in Figure 4, 10 days of repeated restraint stress (stressed group) significantly elicited depression in mice with increased immobility. Paroxetine, a commonly used clinical anti-depressant drug, significantly inhibited the immobility. Simultaneously, treatment with AEO significantly and dose-dependently decreased the immobility, which effect (20 and 40 mg/kg) is comparable to that of paroxetine (10 mg/kg).

### 2.5. Effects of AEO on Restraint Stress-Induced Depression by the Forced Swimming (FS) Test in Mice

**Figure 5:**
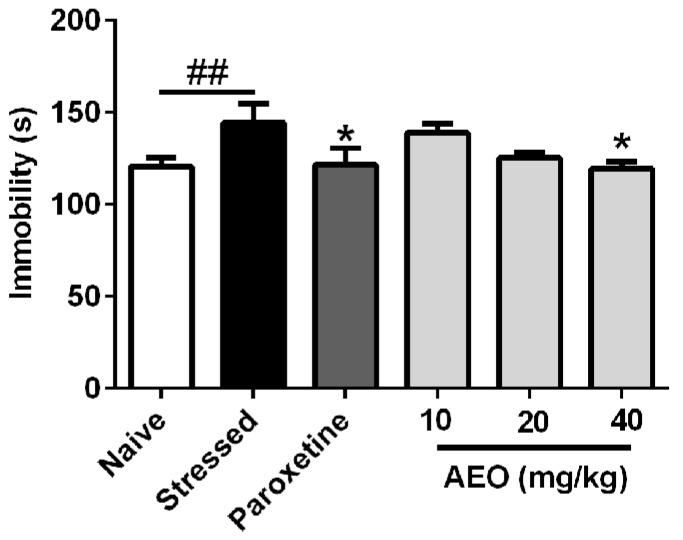
Effects of agarwood essential oil (AEO) on restraint stress-induced depression as tested by the forced swimming test. All mice except for the naive group received 10 days restraint stress, and mice immobility was recorded during a 4 min test period. Each value represents mean ± SEM with *n* = 12, ^##^ *p* < 0.01 vs. the naive group, * *p* < 0.05 vs. the stressed group.

As shown in Figure 5, the forced swimming (FS) test illuminated that ten days of repeated restraint stress (stressed group) significantly increased the immobility of mice. Paroxetine (10 mg/kg) and AEO significantly inhibited the mice immobility. 40 mg/kg AEO showed the best efficiency among the tested dosages with a comparable effect to that of paroxetine (10 mg/kg).

### 2.6. Effects of AEO on Inflammatory Cytokines in Restraint Stress-Induced Mice Serum

**Figure 6:**
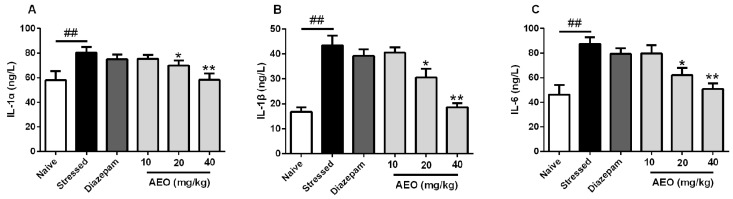
Effects of AEO on the levels of (**A**) interleukin 1α (IL-1α), (**B**) IL-1β, and (**C**) IL-6 in the serum of restraint stress-induced mice. Each value represents mean ± SEM with *n* = 8, ^##^ *p* < 0.01 vs. the naive group, * *p* < 0.05 and ** *p* < 0.01 vs. the stressed group.

It has been reported that prior exposure to repeated restraint enhances the homotypic stress-induced increase in cytokines in plasma, which has a close relationship with the HPA hyperactivity [21]. Our previous investigation found that agarwood could inhibit cytokines in the serum of mice (data not shown). Therefore, we assessed the effects of AEO on inflammatory cytokines in mice with anxiety and depression induced by restraint stress. As the results show in Figure 6, treatment with AEO dose-dependently decreased the levels of IL-1α, IL-1β, and IL-6, in which 40 mg/kg AEO showed the best efficiency among the tested dosages, whereas 10 mg/kg AEO did not display an apparent inhibitive effect.

### 2.7. Effects of AEO on nNOS Gene Transcription and Protein Expression in Mice Brain

**Figure 7:**
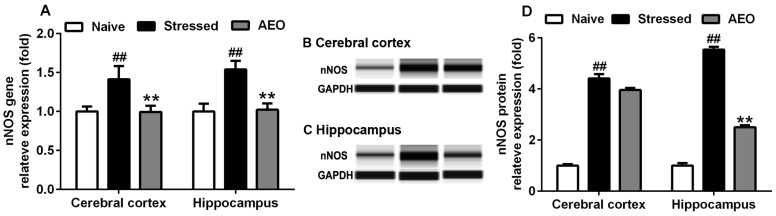
Effects of AEO on (**A**) neuronal nitric oxide synthase (nNOS) gene transcription and (**B**) protein expression in the cerebral cortex and (**C**) hippocampus of mice. (**D**) The quantitative results of relative protein expression are shown in fold. Each value represents mean ± SEM, *n* = 3 with three independent biological replicates, ^##^ *p* < 0.01 vs. the naive group, ** *p* < 0.01 vs. the stressed group.

The HPA axis could not only be influenced by IL concentration, but also the NO level. NO is produced intracellularly by NOS, a complex protein with three isoforms, including nNOS, endothelial NOS (eNOS, and iNOS. nNOS, the first isoform to be purified and cloned from the brain, is considered to be responsible for NO production in neurons [26,27]. The results, as shown in Figure 7, revealed that repeated restraint stress (stressed group) increased nNOS gene and protein expression in the cerebral cortex and hippocampus. Treatment with AEO (40 mg/kg) significantly inhibited the mRNA levels of nNOS in the cerebral cortex and hippocampus. Concomitant measurement of protein expression by a capillary immunoassay (Simple Western) indicated that administration of AEO significantly inhibited the nNOS protein level in the hippocampus but had an unobvious effect in the cerebral cortex.

### 2.8. Effects of AEO on CRF and CRFR Expression in Mice Brain

**Figure 8:**
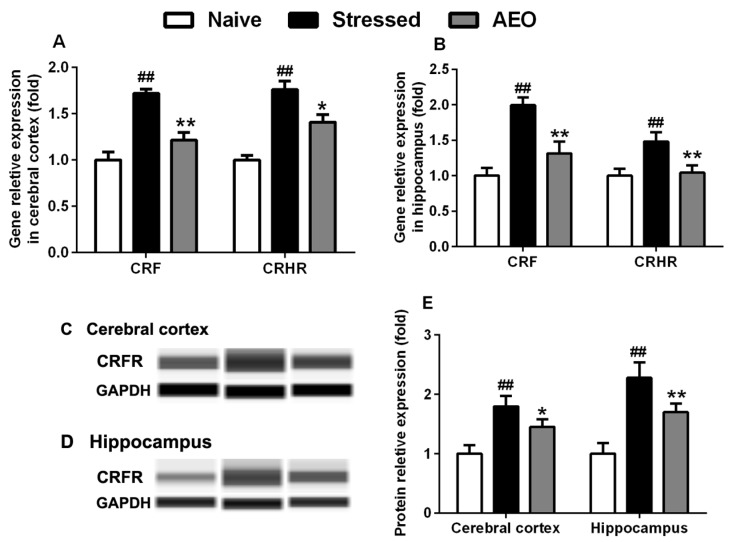
Effects of AEO on the expression of corticotropin releasing factor (CRF) and CRF receptor (CRFR). CRF and CRFR gene transcription were assessed by RT-PCR in (**A**) cerebral cortex and (**B**) hippocampus, and CRFR protein expression was detected by Wes in the (**C**) cerebral cortex and (**D**) hippocampus of mice. (**E**) The quantitative result of relative expression was showed in fold. Each value represents mean ± SEM, *n* = 3 with three independent biological replicates. ^##^ *p* < 0.01 vs. the naive group, * *p* < 0.05 and ** *p* < 0.01 vs. the stressed group.

Almost 20 different peptide systems have been suggested to have roles in the modulation of anxiety, of which CRF, one of the most intensively studied neuropeptides, and is the major physiological regulator of the stress response [19]. We assessed the effects of AEO on CRF and its associated receptor, CRFR, in mice cerebral cortex and hippocampus. The results showed that AEO significantly decreased the gene expression of CRF in the cerebral cortex and hippocampus. Simultaneously, treatment with AEO significantly inhibited the gene transcription and protein expression of CRFR in the cerebral cortex and hippocampus of mice (Figure 8).

### 2.9. Effects of AEO on ACTH and CORT Concentrations in the Serum of Restraint Stress-Induced Mice

**Figure 9:**
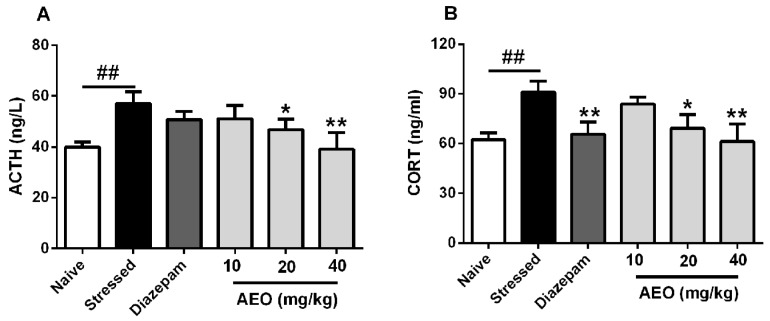
Effects of AEO on (**A**) adrenocorticotropic hormone (ACTH) and (**B**) corticosterone (CORT) concentrations in the serum of restraint stress-induced mice. Each value represents mean ± SEM with *n* = 8, ^##^ *p* < 0.01 vs. the naive group, * *p* < 0.05 and ** *p* < 0.01 vs. the stressed group.

Based on the above results, we checked the key nodes downstream of the HPA axis, including ACTH and CORT. As the results shown in Figure 9, administration of AEO significantly and dose-dependently decreased the levels of ACTH and CORT in serum of mice, whereas diazepam significantly decreased the CORT concentration and had no apparent effect on ACTH.

## 3. Discussion

This study demonstrated that AEO, derived from the whole tree agarwood-inducing technique, possesses anxiolytic and antidepressant effects on restraint stress-induced mice through multiple animal behavior tests. Its potential mechanism is related to the inhibition of CRF and its pathway, the hyperactive HPA axis.

With the quickening pace of modern life and the increasing professional competition, the frequency of anxiety and depression is increasing, and stress might play a vital important role in the pathogeneses of anxiety and depression. Restraint has been extensively used to study the role of stress in psychopathology, especially on anxiety and depression [28]. Therefore, we applied the restraint stress model elicited by a 3-h restraint per day for a successive 10 days to induce anxiety and depression. The results showed that 10-successive days of restraint stress led to the occurrence of anxiety and depression behaviors in mice. Treatment with AEO significantly increased the time, distance, and entries in open arms as assessed by the EPM test. Additionally, AEO dose-dependently increased the time, distance, and transition in light compartment by the LDE test. The OF test also indicated that AEO had anxiolytic effect with the time and distance increase in central area. Additionally, AEO had a significant antidepressant effect as shown by decreased immobility in the TS and the FS test.

Our previous experiments found that AEO has an anti-inflammatory effect which could inhibit the level of cytokines. Cytokines, immune substances excreted by immunocytes, can also be secreted by non-immunocytes and act as messengers between neuroimmune cells [21]. Interleukins such as IL-1α, IL-1β, and IL-6 are the most important ones [29], and can be elevated in serum during stress-related conditions [30]. It has been reported that restraint stress can increase the concentration of IL-1β in the cerebral cortex, hippocampus, hypothalamus, and serum [21]. Therefore, the concentrations of cytokines, including IL-1α, IL-1β, and IL-6, were detected by ELISA kits in the serum of mice with restraint stress-induced anxiety and depression. As the results show in Figure 4, administration of AEO significantly and dose-dependently inhibited the concentrations of IL-1α, IL-1β, and IL-6. It was reported that IL-1α can activate the HPA axis, leading to the synthesis and release of CRF, ACTH, and CORT [29]. Simultaneously, IL-1β, secreted by brain, pituitary, and adrenal cortex, has an influence on CRF release from hypothalamus, ACTH secretion from pituitary, and adrenocortical hormone excretion from adrenal cortex [21]. IL-1β could also directly induce ACTH secretion through IL-1 type 1 receptor (IL-1R1) [31].

In general, NO is responsible for endothelium-derived relaxing factor activity in blood vessels and formed in macrophages and neutrophils in response to endotoxin. However, NO is also an important neurotransmitter, participating in the regulation of the HPA axis. Under physiological status, nNOS is expressed as a structural constitution and is the main species of NOS. When stimulated by cytokines or stress, the expression of iNOS can be up-regulated, catalyzing the formation of NO [32], which participates in the maincenter-induced release of CRF and plays an important role in the regulation of the HPA axis. Furthermore, activation of HPA axis by IL-1β and upregulation of nNOS expression occur at the same time in the cerebral cortex and hippocampus [33]. Treatment with AEO not only inhibited the concentration of IL-1β in serum, but also decreased the expression of nNOS in the cerebral cortex and hippocampus.

CRF, a kind of neuropeptide, can be influenced by cytokines and NO. It is the upstream regulator of HPA axis [34,35], and has attracted extensive attention worldwide recently as a result of its anxiolytic and antidepressant effects [22,36]. The RT-PCR and Western Blot results found that AEO significantly inhibited the gene expression of CRF in the cerebral cortex, and also significantly decreased the gene and protein expression in cerebral cortex and hippocampus. In order to verify the effects of AEO on CRF, we investigated the downstream factors of the HPA axis, such as ACTH and CORT. The results showed that administration of AEO significantly decreased the levels of ACTH and CORT in the serum of restraint stress-induced mice.

Above all, these results demonstrated that the anxiolytic and depressant effects of AEO are related to the inhibition of CRF and its pathway, the hyperactive HPA axis, which might have promising utility in the prevention and treatment of anxiety and depression.

## 4. Materials and Methods

### 4.1. AEO Preparation and Chemical Analysis

The agarwood, induced by the whole tree agarwood–inducing technique [37], was purchased from Huazhou county (Guangdong province, China) and was identified by Prof. Jian-He Wei. The AEO was prepared and chemical analyzed as previously reported [14]. Briefly, the AEO was extracted by hydrodistillation for 12 h, dried over anhydrous sodium sulfate (Na_2_SO_4_), and stored in a freezer at −20 °C. A gas chromatograph (GC) equipped with a HP-5MS capillary column (5% phenylmethylsiloxane, 30 m × 0.25 mm i.d., film thickness 0.25 μm) and a mass spectrometer (MS) with an ion trap detector (Agilent Technologies, Santa Clara, CA, USA) in full scan mode under electron impact ionization (70 eV) were used to analyze the chemical components of AEO. The identification was completed by comparing their mass spectra with those stored in the NIST 11 database and MSD Chemstation. The results showed that 68 compounds, representing 98.244% of the AEO, were identified, in which 34 sesquiterpenes were the main (51.132%) components, alongside with 13 aromatic compounds (24.114%) and 21 other known compounds (19.823%).

### 4.2. Animals

Adult male Institute of Cancer Research (ICR) mice, weighing 18–20 g, were provided by the Vital River Laboratories (Qualified No. SYXK 2016-0011, Beijing, China). All animals were housed in plastic cages in a controlled environment at the temperature of 23 ± 2 °C and 60 ± 5% humidity with an alternating 12 h light, 12 h dark cycle, with access to water and diet *ad libitum*. All behavioral evaluations were performed during the day (8:00 a.m. to 8:00 p.m.). The animal experiments were performed in accordance with the NIH Guide for the Care and Use of Laboratory Animals and performed under the approval and supervision of the Animal Ethics Committee of the Institute of Medicinal Plant Development, Chinese Academy of Medical Sciences (Registration Number: #IMPLAD2017030715, Approval Date: 7 March 2017).

### 4.3. Reagents

Dimethyl sulfoxide (DMSO) was bought from Sigma (St. Louis, MO, USA). Enzyme-linked immunosorbent assay (ELISA) kits were bought from Jiancheng Bioengineering Institute (Nanjing, China) and were used according to the manuals’ instructions. Diazepam and paroxetine were purchased from the National Institute for Food and Drug Control (Beijing, China) and prepared in saline consisting of 1% Tween 80. AEO was first dissolved in DMSO at the concentration of 0.5 g/mL, and then suspended in saline containing 1% Tween 80 based on doses of 10, 20, and 40 mg/kg. All drugs were administrated intraperitoneally at a dose of 10 mL/kg.

### 4.4. Experimental Design

To investigate the anxiolytic and depressant effects of AEO on restraint stress-induced mice model, the animals were divided into seven groups (*n* = 12 per group): naive, restraint stressed, restraint stress treated with diazepam (2.5 mg/kg), paroxetine (10 mg/kg), and AEO (10, 20, and 40 mg/kg). To elicit restraint stress, all the mice, except for the naive group, were individually placed into a tube (diameter: 3 cm, length: 12.5 cm) at set times daily from 11:00 a.m. to 2:00 p.m. for 3 h on 10 consecutive days. After the exposure to restraint stress, mice were removed from the tube and returned to their original cage. Naive mice were undisturbed in their original cages. The drugs, including AEO, diazepam, and paroxetine, were suspended in saline solution containing 1% Tween 80, and injected intraperitoneally before restraint stress administration. The naive and stressed groups were treated intraperitoneally with saline solution and saline solution containing 1% Tween 80, respectively. After 10 days treatment, animal behavior tests were used to assess the anxielytic and antidepressant effects of AEO. After the completion of the behavior tests, the mice were sacrificed. The blood samples and brain tissues were rapidly collected. The blood samples were centrifugated at 2500 rpm for 15 min. The cortices and hippocampi were dissected on ice.

### 4.5. Elevated Plus Maze Test

The elevated plus maze test (EPM) apparatus consists of two opposed open arms (30 cm × 10 cm) and two opposed closed arms (30 cm × 10 cm × 20 cm) mounted at an angle of 90°, all facing a central platform (7.5 cm × 7.5 cm) elevated 70 cm above the ground. This test has been widely validated for the investigation of anxiety in rodents [38]. Mice were initially placed at the center of the platform facing one of the open arms. The time spent, distance moved, and number of entries in the open arms was recorded during the 5 min exploration.

### 4.6. Light Dark Exploration Test

The light dark exploration test, which is based on the innate aversion of rodents to brightly lit areas and on the spontaneous exploratory behavior in response to a novel environment and light [39], is a sensitive model for the detection of activity in disorders related to anxiety [25]. The apparatus consists of a rectangular box (20 cm × 12 cm × 12 cm), divided equally into a dark compartment and an illuminated compartment with an opening (4 cm × 4 cm) between the division zones. Both compartments possess 40 infrared-emitting diodes and an infrared camera to detect the movement of tested animals. The apparatus is connected to a computer to record the time spent, distance moved, and number of transitions in each compartment during a 5 min session. Animals were placed in the center of the light area facing away from the opening.

### 4.7. Open Field Test

The open field test evaluates the general locomotor and exploratory behaviors of mice. A computer-aided controlling system consisting of four cages (35 cm × 35 cm × 30 cm, length × width × height) with a 120 Lux light source and a video camera at the top was used to record mice activity as previously reported [40,41]. The animals were placed in the cages individually for 2 min of acclimatization. The movements of mice were recorded for 10 min using cameras with a computer-aided image processing system. The selected movement threshold was 6.5 cm/s. The time spent and distance moved in the central area was collected to reveal the anxiolytic effect of AEO.

### 4.8. Tail Suspension Test

The tail suspension test was carried out as the previous report with a little modification [42]. A computer-aided controlling system consists of eight suspension units divided by walls. Each mouse was suspended by the tail using an adhesive tape for 6 min, and the immobility time during the final 4 min was recorded automatically by the software.

### 4.9. Forced Swimming Test

The FST was carried out on mice according to the method of Porsolt [43]. Briefly, mice were individually placed into a plastic cylinder (20 cm in height, 18 cm in diameter) filled with 12 cm high water (24 ± 1 °C). All animals were forced to swim for 6 min, and the immobility time during the final 4 min interval of the test was recorded by a computer-aided controlling system. Immobility time was defined as the time spent by the mouse floating in the water without struggling, and making only those movements necessary to keep its head above the water.

### 4.10. RT-PCR

The mRNA levels of nNOS, CRF, and CRFR were investigated using real-time polymerase chain reaction (RT-PCR). Total RNA extraction, cDNA synthesis, and quantitative PCR assays were performed as previously described [44]. Samples were cycled 40 times using a Bio-Rad C1000 Thermal cycler (Bio-Rad CFX96 Real-Time System, Hercules City, CA, USA). Bio-Rad C1000 cycle conditions were as follows: 4 min at 95 °C followed by 40 cycles of 15 s at 95 °C, 20 s at 60 °C, and 40 s at 72 °C. The cycle threshold (CT) was calculated under default settings using real-time sequence detection software (Bio-Rad CRX Manager). At least three independent biological replicates were performed to ensure the reproducibility of the data. The gene-specific primers used for quantitative PCR are listed in Table 1.

### 4.11. Western Blot Experiment

The protein levels of nNOS and CRFR were investigated by capillary immunoassay (Simple Western) as previously reported [45,46]. Protein was extracted from the cerebral cortex and hippocampus of mice using a RIPA buffer (CWBio, Beijing, China). The protein concentration was determined by the bicinchoninic acid (BCA) assay (Biomiga, San Diego, CA, USA). The Wes Separation 12–230 kDa Capillary Cartridges Kit (SM-W004) was used for all Simple Western experiments on the ProteinSimple Wes system (ProteinSimple, Santa Clara, CA, USA). 3 μL of protein extract from each sample was loaded to the kit at the final concentration of 1.5 mg/mL. nNOS, CRFR1, and GAPDH (loading control) were detected by primary antibodies bs-10197R (1:50, Bioss, Beijing, China), NBP1-00175 (1:100, NOVUS, Littleton, CO, USA) and bs-2188R (1:100, Bioss, Beijing, China). Goat Anti-Rabbit Secondary HRP Conjugate (ready-to-use reagent) and Rabbit Anti-Goat Secondary HRP Conjugate (1:50, EASYBIO, Beijing, China) were used. Default running and detection programs were used across all assays (separation time 30 min, separation voltage 375 Volts, antibody diluent time 5 min, primary antibody time 30 min, secondary antibody time 30 min). Compass software (ProteinSimple, Santa Clare, CA, USA) was used to collect the chromatograms data and visualize the virtual gels. Relative protein quantification was generated from chromatograms of the indicated samples and showed in simulative blot bands. Three independent biological replicates were performed to ensure the reproducibility of the data.

### 4.12. Statistics Analysis

All the results were firstly tested in normal distribution and variance homogeneity, and the abnormal data was eliminated. Then a one-way analysis of variance (ANOVA) followed by Dunnett’s test was used by SPSS 17.0 (Chicago, IL, USA). Significance was accepted at *p* < 0.05 and the data are expressed as mean ± SEM.

## Figures and Tables

**Table 1:** Primers used in quantitative real-time polymerase chain reaction (RT-PCR) analysis.

Name	Forward (5′-3′)	Reverse (3′-5′)
nNOS	CCGATCATTGACGGCGAGAAT	CTGGTGAAGGAACGGGTCAG
CRF	CCTCAGCCGGTTCTGATCC	GCGGAAAAAGTTAGCCGCAG
CRFR	GGGCAGCCCGTGTGAATTATT	ATGACGGCAATGTGGTAGTGC
β-actin	GGCTGTATTCCCCTCCATCG	CCAGTTGGTAACAATGCCATGT
